# Orthogonal inactivation of influenza and the creation of detergent resistant viral aggregates: towards a novel vaccine strategy

**DOI:** 10.1186/1743-422X-9-72

**Published:** 2012-03-26

**Authors:** Julie M Belanger, Yossef Raviv, Mathias Viard, Ulrich Baxa, Robert Blumenthal

**Affiliations:** 1 Center for Cancer Research Nanobiology Program, National Cancer Institute Frederick, Frederick, USA; 2 Basic Research Program, SAIC-Frederick, Inc., NCI-Frederick, Frederick, Maryland 21702, USA; 3 Advanced Technology Program, SAIC-Frederick, Inc., NCI-Frederick, Frederick, Maryland 21702, USA; 4 Department of Chemistry and Physics, King's College, Wilkes-Barre, PA, USA

**Keywords:** Influenza virus, Detergent, Detergent resistance, Hemagglutinin, Triton, Vaccine, Inactivation, Human immunodeficiency virus, HIV-1, Orthogonal

## Abstract

**Background:**

It has been previously shown that enveloped viruses can be inactivated using aryl azides, such as 1-iodo-5-azidonaphthalene (INA), plus UVA irradiation with preservation of surface epitopes in the inactivated virus preparations. Prolonged UVA irradiation in the presence of INA results in ROS-species formation, which in turn results in detergent resistant viral protein fractions.

**Results:**

Herein, we characterize the applicability of this technique to inactivate influenza. It is shown that influenza virus + INA (100 micromolar) + UVA irradiation for 30 minutes results in a significant (*p *< 0.05) increase in pelletablehemagglutinin after Triton X-100 treatment followed by ultracentrifugation. Additionally, characterization of the virus suspension by immunogold labeling in cryo-EM, and viral pellet characterization via immunoprecipitation with a neutralizing antibody, shows preservation of neutralization epitopes after this treatment.

**Conclusion:**

These orthogonally inactivated viral preparations with detergent resistant fractions are being explored as a novel route for safe, effective inactivated vaccines generated from a variety of enveloped viruses.

## Background

Universal techniques for the rapid generation of safe, effective vaccines from a variety of viruses are needed to protect against a host of diseases. Inactivated virus vaccines, which start with infectious material, can be produced through routes such as chemical inactivation. These types of vaccines are currently being used (such as in the USA for Influenza), but continue to have limitations, such as the inability to cross-react between viral strains and subtypes. For emerging or ill-characterized novel pathogens, inactivated vaccines raise the serious concern of safety. It is generally agreed that the required inactivation of virus for use in such vaccines is at least 15 logs of inactivation [1]. Such a high level of inactivation is difficult to determine, and usually relies on the use of the combination of techniques for safe inactivation. These techniques typically operate on mechanisms independent of one another, and are considered "orthogonal" inactivation steps. It is generally accepted that the additive effect of these steps is used towards the "15 logs" of inactivation suggested for the generation of a safe vaccine.

One typical method for orthogonal inactivation, is the generation of "split virus" vaccines. These types of vaccines, currently used in some influenza preparations, rely on chemical means for the initial inactivation of the virus, followed by detergent treatment to "split" or solubilize the virus. This solubilized viral protein preparation is then further purified to obtain a specific protein (hemagglutinin in the case of influenza). This purified protein only represents a fraction of the overall native virion, and has been removed from its native environment in the viral membrane.

A more idealized method for such vaccines, would apply orthogonal inactivation techniques (ie chemical inactivation + detergent) for safety, but maintain the epitopes that represent the native virion in order to elicit a more effective immune response similar to whole virus vaccines [2]. Additionally, such an idealized vaccine candidate could be imagined to contain preserved epitopes that expose functionally conserved regions for the generation of cross-reactive neutralizing antibodies [3]. In the context of a "split virus" vaccine, if the chemically inactivated virus was partially detergent resistant before the detergent treatment step, then the detergent treatment step would serve solely to mop-up any live viruses, while leaving the inactivated virus similar to the native virion. Subsequent purification could then be used to isolate the intact inactivated virus instead of solubilized proteins.

One way to achieve this is to use a chemical inactivating agent that is specific to the hydrophobic region of the bilayer membrane in enveloped viruses, namely 1-iodo-5-azidonaphthalene (INA) [4]. This hydrophobic probe, when activated by UVA irradiation for 2-5 minutes, results in the inactivation of a variety of enveloped viruses with preservation of surface epitopes [5-9]. Additionally, in the case of HIV-1, it was found that prolonged UVA irradiation (15 minutes) in the presence of INA resulted in reactive oxygen species (ROS) generation, that caused detergent resistance of various viral proteins, with the preservation of epitopes recognized by neutralizing antibodies [8,10]. These detergent resistant fractions were thought to contain fragments of virus similar to the native virion, and not completely solubilized proteins.

This technique can potentially be applied to a variety of enveloped viruses for the production of safe inactivated vaccines that are orthogonally inactivated and contain viral fragments similar to the native virus. Herein, we explore the applicability of this technique to the inactivation of influenza virus, with particular attention to the characterization of the detergent resistant fraction and the preservation of neutralization epitopes. This is a necessary step towards the demonstration of this novel approach as a "universal" and safe method for the generation of inactivated envelope virus vaccines.

## Results and discussion

It has been previously shown that the treatment of enveloped viruses with aryl azides and minimal UVA irradiation (for 2 minutes) results in complete viral inactivation due to covalent binding of the hydrophobic azido compound within the bilayer of enveloped viruses [5-9]. These inactivated virus preparations have shown to be effective as vaccine candidates in animal studies [5,6]. More recently, we have shown that with prolonged UVA irradiation (15 min irradiation time) in the presence of aryl-azido compounds, protein aggregates can be induced in HIV-1, an enveloped virus, as a result of reactive oxygen species formation [8]. This ROS-modification has been shown to induce detergent resistance in key viral proteins, which makes this treatment applicable towards the generation of a novel type of orthogonally inactivated vaccine strategy [10].

Here, we extend this finding to another enveloped virus, influenza, to show the "universal" and general nature of this method to treat different enveloped viruses. We show that for two different strains of Influenza A virus, X31 (an H3N2 virus) and PR8 (an H1N1 virus), a similar trend of inactivation and detergent resistance is seen, characterized herein using Western blot, ultracentrifugation, MDCK plaque assays, hemagglutination assay, immunoprecipitation, and cryo-electron microscopy.

### Treatment of influenza with INA plus prolonged UVA irradiation results in viral protein aggregation and minimal effect on hemagglutination

**Figure 1:**
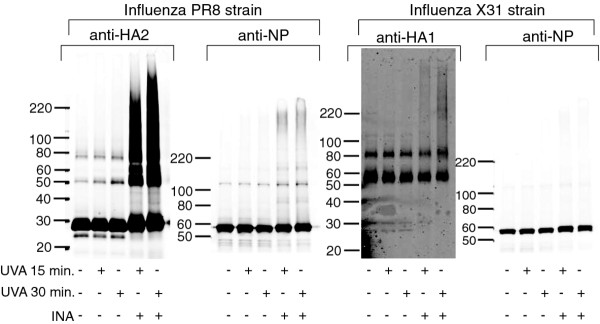
**Influenza proteins as visualized by Western blot after UVA irradiation with and without INA**. Two strains of influenza PR8 (H1N1 virus) and X31 (H3N2) were tested with varying treatments as specified in the figure. Western blots were subsequently probed with the antibody noted, either anti-HA2, anti-HA1 or anti-NP. The anti-NP was cross reactive to both strains, whereas anti-HA2 and anti-HA1 were strain specific. Note that the UVA samples also contain DMSO added in the same amount needed for the addition of the INA to the INA-treated samples.

Similar to previous results as seen with HIV [10], it was found that the viral proteins of the influenza virus can be modified via reactive oxygen species (ROS) induced as a by-product of prolonged UVA irradiation in the presence of an aryl azide. Consistent with the previous findings for HIV, the transmembrane protein of Influenza (HA2) forms aggregates upon aryl-azide + UVA treatment, whereas the external proteins (HA1 in influenza) remain largely unaffected (see Figure 1). This amount of aggregation is increased when the samples were treated with UVA irradiation for 30 minutes versus 15 minutes.

In contrast with previous findings (with HIV capsid protein), the solubility of the nucleoprotein (NP) of influenza virus remains unaffected by ROS species, and Western blot shows only the main NP band (no protein aggregates). The nucleoprotein antibody used was cross-reactive to both strains, and shows that both strains of influenza tested do not exhibit ROS-induced NP protein aggregation, as seen in Figure 1. This may be due to the nature of the nucleoprotein and its assembly in the viral core. The nucleoprotein inter- or intra- protein interactions may be such that the ROS species are unable to penetrate and modify the proteins, or it is possible that the chemical product responsible for the ROS generation (from the prolonged irradiation of the aryl azide) may not penetrate these proteins. This hypothesis is further supported by the detergent treatment experiments described in the next section, and is further elaborated therein.

In addition to this Western blot analysis, the ability of the virus to agglutinate red blood cells was tested before and after INA treatment, with the results given in Table 1. This assay indicates that the hemagglutinin (HA) protein located on the surface of the influenza maintains its ability to establish a binding network that prevents the settling of the RBC's even after treatment with prolonged UVA irradiation in the presence of INA. While there is some damage as indicated by the fold reduction in HAU titer for INA treatments after UVA irradiation for 15 and 30 min., the virus still maintains the ability to agglutinate red blood cells, albeit at lower dilutions.

**Table 1:** Results of Hemagglutination assay using X31 after various treatments

Treatment	Strain, lot # ending	Fold reduction in HAU titer*
Flu + INA + UVA 5 min.	X31, 0627	0

	X31, 1203A	0

Flu + INA + UVA 15 min.	X31, 0627	0

	X31, 1203A	2

Flu + INA + UVA 30 min.	X31, 0627	4

	X31, 1203A	4

Each experiment done in duplicate

*relative to untreated control

### Viral proteins exhibit detergent resistance

**Figure 2:**
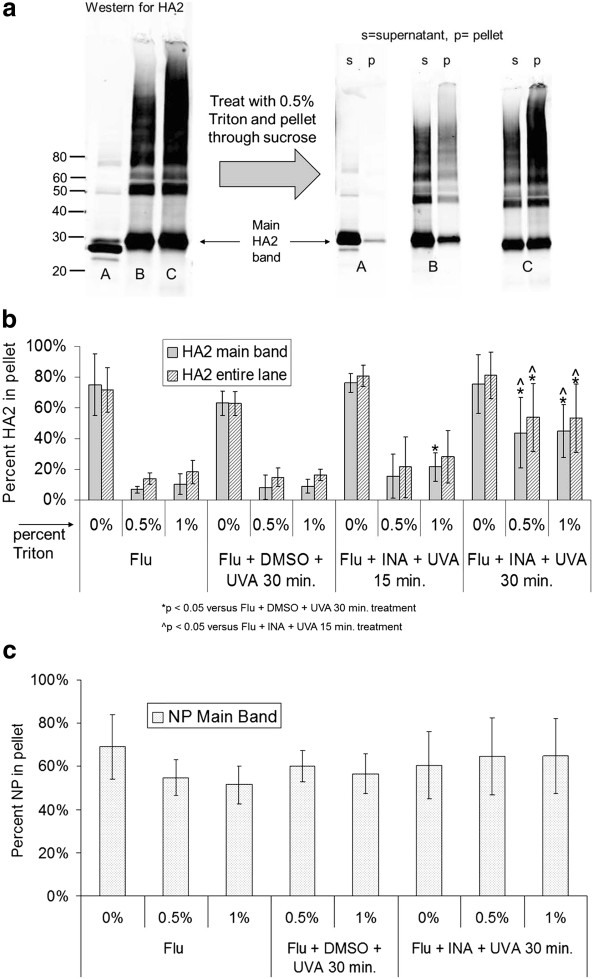
**Increase in the pelletable fraction of influenza PR8 hemagglutinin (HA2) after treatment of virus with INA + UVA irradiation**. **a) **side-by-side comparison of the raw data used to determine percent HA2 in pellet showing an increase in the pelletable main band as seen by eye, where A = untreated influenza, B = influenza + INA + UVA 15 min., C = influenza + INA + UVA 30 min., **b) **quantitation of the percentage of HA2 in the pelleted fraction after the treatments as specified, **c) **quantitation of influenza nucleoprotein (NP) in the pelleted fraction. Data for **b) **and **c) **is from the integration of the entire lane in Western blot, except for those labeled "main band only" data, which represents data obtained from the integration of the main protein band (either HA2 or NP) alone (as outlined in Materials and Methods). All data was performed in true triplicate, and error bars represent plus and minus two standard deviations. P-values were calculated using a two-tailed student's T-test with unequal variances, with significant values (*p *< 0.05) as denoted below the figure.

It has been previously shown that INA treatment of influenza and other enveloped viruses results in viral inactivation after 2-5 minutes of irradiation [5-9] and therefore prolonged UVA irradiation to induce ROS species is not necessary for viral inactivation. Additionally, according to Table 1 above, the ability of the influenza virus to agglutinate red blood cells is also maintained after this brief treatment to inactivate the virus. However, the protein aggregation that is seen via Western blot as a result of prolonged irradiation (UVA irradiation of 15 minutes or more) has been shown to correlate with detergent resistance in another enveloped virus, HIV-1 [10]. This "detergent resistance" is being explored as a novel method for the preparation of an orthogonally inactivated virus vaccine using aryl azides and detergent treatment, as initially proposed for HIV. To this end, the induced "detergent resistance" of two viral proteins in influenza was studied: HA2 protein (the transmembrane unit of hemagglutinin) and NP (nucleoprotein, an internal protein in the virus). Detergent resistance here is defined as the inability to solubilize a viral protein after treatment with either 0.5% or 1% Triton X-100. This can be quantified via ultracentrifugation through a sucrose pad, and analyzing the amount of pelletable viral protein via Western blot (as described in Materials and Methods). The results are summarized in Figure 2 below, where an increase in the "% protein in pellet", signifies an increase in the "detergent resistant" fraction.

One major difference between the detergent resistance seen here for influenza, versus HIV-1, is the irradiation time required to exhibit detergent resistance. It can be seen in Figure 2a, for the influenza viral protein, HA2, that when the irradiation time is increased from 15 minutes to 30 minutes in the presence of INA, that the amount of protein aggregation significantly increases, as well as the amount of pelletable non-aggregated ("main band") protein. When the amount of influenza viral protein HA2 was quantified from these and additional Western blots, it was seen that the amount of pelletable protein was increased significantly (*p *< 0.05) after treatment with 100 micromolar INA + UVA irradiation for 30 minutes, but was not significantly increased with INA + UVA for 15 minutes, versus UVA irradiation for 30 minutes alone (without INA). Furthermore, the amount of detergent resistant HA2 was also significantly increased (*p *< 0.05) when 30 minutes of UVA irradiation was used (with INA) over the comparable 15 minute treatment. This was shown to hold true for both the aggregated region of proteins in the Western blot as well as the non-aggregated ("main band") proteins. Additionally, it is known that non-ionic detergents such as Triton X-100, do not readily denature proteins and have been used to isolate proteins while maintaining their function [11]. The ability of this treatment to induce detergent resistance, combined with the "gentle" nature of non-ionic detergent treatment, is of importance if this preparation is to be used towards the generation of a vaccine, with the hypothesis that the viral protein pellet may better represent the intact proteins of the native virion.

Interestingly, when the detergent resistance of NP was studied using the same method after INA treatment + UVA irradiation for 30 minutes, there was no significant increase in the pelleted fraction of the influenza viral protein NP over controls. This finding is in agreement with the aforementioned Western blot analysis (Figure 1) indicating that there is no protein aggregation of NP with INA + UVA treatment. Previously, we reported that there is a correlation between this aggregation as seen in Western blot with the detergent resistance seen [10]. Surprisingly, independently of the treatment performed on the virus, 70% of the total NP within the sample can be pelleted through ultracentrifugation (Figure 2c). This implies that there is some inherent detergent resistance of the structure adopted by NP within the influenza virus. It has been shown that proteins can differ in their solubility by non-ionic detergents (such as the Triton used herein), with stronger detergents (such as ionic detergents) needed in order to break stronger protein-protein interactions [12,13]. It is possible that the interactions between the nucleoproteins in influenza, either intra- or inter- NP interactions, are too strong to be dissociated with Triton X-100. This phenomenon has been seen before, when Triton X-100 solubilization and pelleting of influenza infected cells was attempted, only to find the majority of NP at the bottom of the gradient [14]. Further characterization of this phenomenon was not pursued, but is taken as a positive result, since in all cases there is a significant portion of NP present in the pellet. The presence of NP in combination with the HA2 protein in the pellet may be favorable towards the generation of cross-reactive immune response if this preparation were to be used in vaccines [3,15].

This finding, with the influenza protein NP (with no increase in pelletable material), is in contrast to HIV-1, where an internal viral protein (capsid, p24) showed significant protein aggregation and detergent resistance after INA plus 15 minutes of UVA irradiation [10]. In the case of HIV, p24 was readily solubilized in Triton X-100. This is of important note, since this method (INA + UVA + Triton) is being proposed as a "universal" method for the orthogonal inactivation of enveloped viruses with the promotion of detergent resistance. These results taken together (HA2 and NP for influenza, gp41 and p24 for HIV-1) suggest that detergent resistance promoted by ROS is applicable to a variety of enveloped viruses, with the caveat that the nature of the proteins themselves lends to subtle differences in the effect of the ROS, and therefore the detergent resistance profile, throughout the virus.

### Influenza inactivation using Triton X-100

While this "INA + prolonged UVA" treatment results in viral inactivation with detergent resistance, it was unclear how much of an affect the Triton alone would be expected to have on viral infectivity. It has been suggested that at least 15 logs of inactivation is needed for vaccine candidates to be considered safe for use [1]. Towards this requirement, it is widely accepted that multiple methods of inactivation that operate on mechanisms independent of one another (ie orthogonal techniques) can be combined to achieve this suggested level of inactivation. A recent study [16] found that Triton can be used to safely inactivate pandemic influenza for use in BL2 conditions, with the caveat that their treatment (percent Triton and duration) may not be directly applicable to all systems, so assessment of Triton inactivation in our system was warranted. In that study, they were also required to remove the Triton due to interference with their cell based assay. Fortuitously, the influenza strains used herein were highly infectious, which allowed for inactivation of the purified virus with Triton, followed by dilution before adding to the cells. At such high dilutions, there were no cytotoxic effects on the cells, and plaques could still be counted for the suboptimal Triton treatments using a cell-based plaque assay. At concentrations any higher than these, cytotoxic effects of the MDCK cells were noted (unpublished observations). For this plaque assay, influenza was treated with varying percentages of Triton at two different temperatures. These experiments were performed in duplicate wells, with the entire experiment reproduced in true duplicate (or greater) and the values for the log reduction in infectivity is shown in Table 2. Additional information regarding the viral lots used, number of plaques counted at each dilution, etc., is given in Additional file 1.

**Table 2:** Influenza is completely inactivated by INA treatment and is inactivated to varying degrees by Triton X-100

Treatment^a ^(Triton %, Temperature, etc)	Logs of inactivation^b^
0%, R.T.	0
	0
	0
	0

0%, 37°C	0.36
	0
	0.33
	0.60

0.2%, R.T.	3.43
	3.04

0.2%, 37°C	5.56
	5.32
	5.48
	4.91

0.3%, R.T.	3.67
	3.30

0.3%, 37°C	5.48
	5.30

0.4%, R.T.	3.87
	4.13
	4.26

0.4%, 37°C	5.48
	5.30

0.5%, R.T.	3.88
	4.00

0.5%, 37°C	5.48
	5.30

0%, RT, +INA (100 uM, UVA = 5 min.)	5.56
	4.56

Duration of Triton treatment was 1 hour with periodic vortex mixing, before dilution and addition to MDCK cells for a plaque assay. Additional information is available in supplementary information

^*a*^*R.T. = room temperature. Only last two entries were INA-treated, as denoted*.

^*b*^*Difference in the number of logs calculated from the room temperature control for the same lot number, taking into consideration any additional dilution effects. The limit of detection of the assay is between 5 and 6 logs of inactivation depending on the viral lot used*

In the MDCK assay, the infectivity of the control virus was determined in plaque-forming units (PFU) and found to be 10^7.65 ^PFU/mL and 10^6.56 ^PFU/mL for the lot numbers ending in 0627 and 1203A, respectively. When the control virus was mock-treated at 37°C, along with the detergent-treated controls (using PBS instead of Triton X-100), the infectious titer was found to decrease only slightly to 10^7.15 ^PFU/mL and 10^6.20 ^PFU/mL, respectively. Additional treatments as listed below in Table 2, show varying logs of inactivation depending on the Triton percent and temperature used, with the limit of detection of the assay at 5-6 logs of inactivation.

As shown in Table 2, the amount of Triton needed to remove at least 5 logs of infectivity (limit of the assay) is shown to be 0.5% when treatment is done at 37°C. For almost all the concentrations of Triton X-100 tested, the number of logs of inactivation increased when the samples were treated with Triton at 37°C versus at room temperature. This indicates that the virus is more efficiently solubilized by Triton at higher temperatures, leading to a higher degree of inactivation.

These results, combined with similar results from another group [16], indicate that Triton can be used to successfully inactivate influenza virus. This is of important note when considering Triton (or potentially other non-ionic detergents) for their used in orthogonal inactivation techniques to reach a "safe" level of inactivation. Additionally, the percent Triton required to inactivate the virus in our study (0.5% Triton at 37°C), when combined with the INA treatment, results in detergent resistant fragments as noted in Figure 2. Therefore, not only is this a viable method for orthogonal inactivation, it also results in a preparation that can be pelleted and enriched in HA and NP proteins through induced detergent resistance (Figure 2). While the entire treated suspension could be used as a vaccine candidate, it may be more applicable to purify the preparation via ultracentrifugation, yielding the detergent resistant viral pellet. With this in mind, we further characterized the viral pellet for recognition by known neutralizing antibodies.

### Retention of HA neutralization epitopes in the viral suspension and detergent resistant pellet

**Figure 3:**
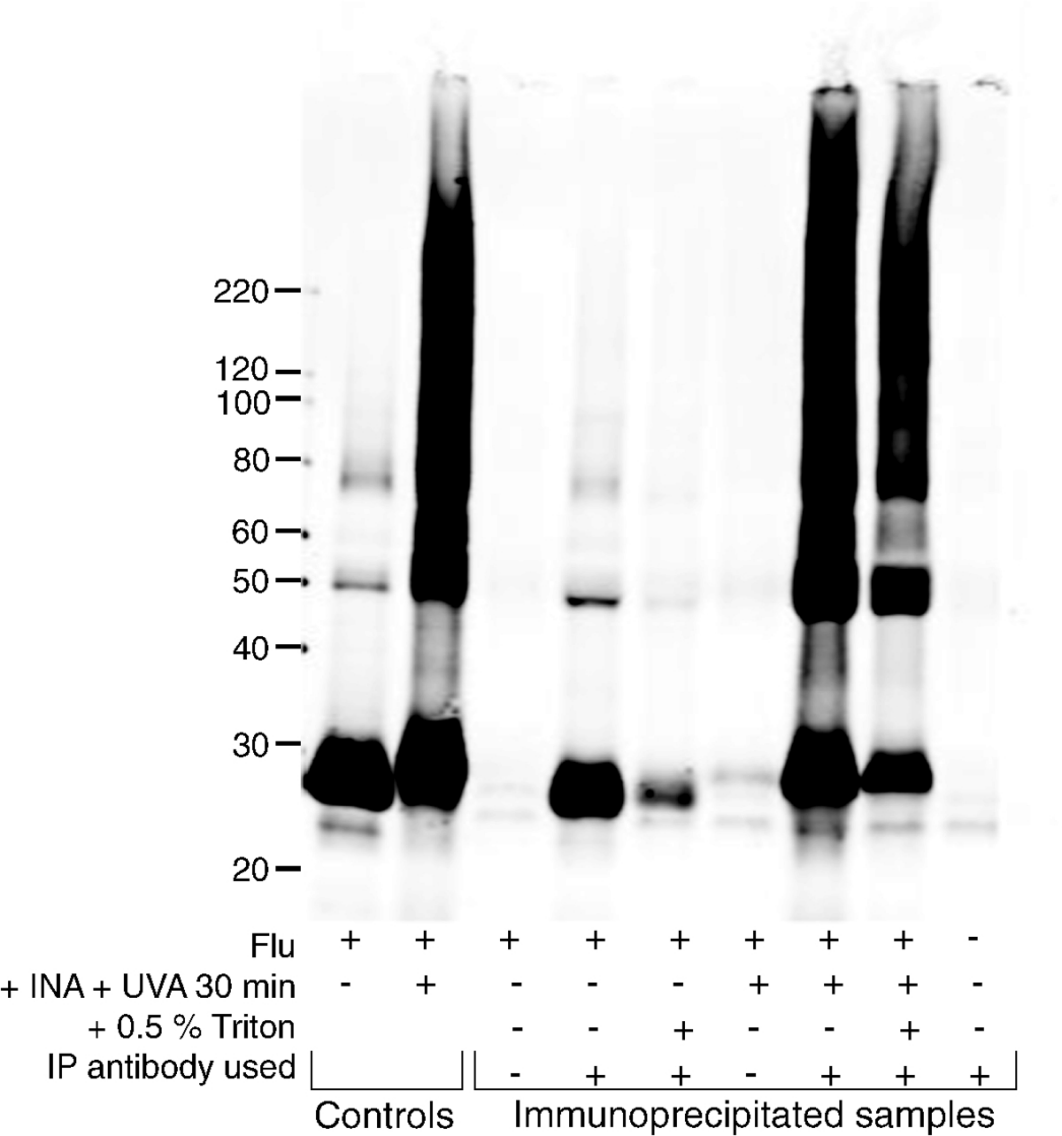
**INA-treatment results in an increase in pelletablehemagglutinin recognized by neutralizing antibodies**. Influenza virus (PR8) was treated as indicated below the figure. "Controls" refers to virus after the treatment specified (not immunoprecipitated). "Immunoprecipitated samples", as indicated, were treated as specified and then pelleted through sucrose. All pellets were then resuspended in 1% Triton and immunoprecipitated using an anti-HA antibody. Mock-IP was also performed without the addition of antibody to assess background binding to the beads. Western blot was probed using anti-HA2. Image shown is representative of duplicate experiments.

To further characterize the detergent resistant viral pellet and the entire viral suspension after treatment with INA plus UVA irradiation for 30 minutes, both immunoprecipitation and cryo-electron microscopy (cryo-EM) were performed. To assess the preservation of neutralization epitopes in the detergent resistant viral pellet, the virus was treated with INA + UVA for 30 minutes followed by detergent treatment, and pelleting. The pellet was then gently resuspended in 1% Triton and immunoprecipitated using an anti-HA antibody that has been shown to neutralize influenza and to inhibit hemagglutination in a variety of influenza A subtypes [17,18]. A Western was then performed on the immunoprecipitated sample using an antibody against HA2 (see Figure 3).

The amount of pelletable HA2 not only is increased in pelleting studies (as shown in Figure 2), but also is shown in Figure 3 to be preserved in the pellet in immunoprecipitation studies. The antibody used for IP was a polyclonal antibody raised against HA, while the antibody used for the Western blot was more specifically against the transmembrane protein HA2. In Figure 2, the pellet obtained for the control sample (flu + Triton alone) did not contain any intact HA as recognized by the IP experiment. Conversely, the sample pelleted after INA + UVA irradiation still contained recognizable hemagglutinin not only in the main band, but also in protein aggregates of higher molecular weight.

**Figure 4:**
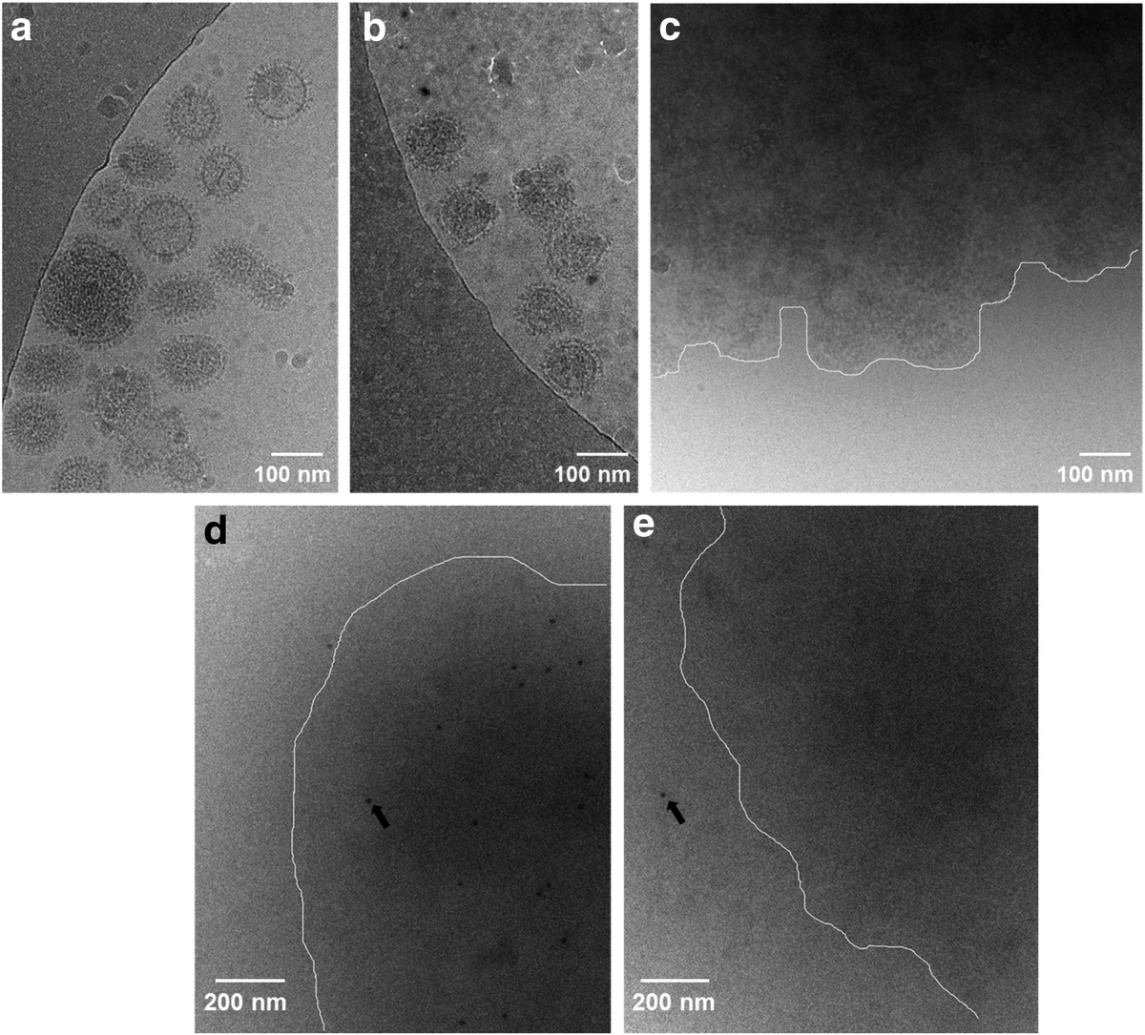
**Cryo-electron microscopy shows intact virions after INA treatment and recognizable hemagglutinin epitopes following Triton treatment**. Influenza (PR8) suspensions were treated with INA + UVA for 30 minutes, then treated with 0.5% Triton X-100 for 1 hour, then fixed and frozen for cryo-EM. **(a) **control influenza, **(b)**influenza + INA + UVA for 30 minutes, **(c) **"b" plus Triton, **(d)**immunogold labeling of virus + INA + UVA + Triton, **(e) **Control immunogold sample same as "d" without primary antibody to assess non-specific binding of secondary immunogold. White lines are drawn to guide the eye in panels "c", "d" and "e", indicating the approximate boundary between the aggregated viral proteins and the bare grid. Arrows indicate immunogold location, in panel "d" within the aggregate, and panel "e" outside the aggregate. Images shown are representative of sections of overall grid.

To ascertain what was happening in the entire treated viral suspension itself after these various treatments, cryo-EM was performed. Cryo-EM allowed for visualization of the viral suspension in its "native" state, without the necessity for pelleting and probing through multistep microbiological techniques (ie IP and Western blot). The influenza virus itself (untreated) is studded with hemagglutinin protein spikes, making it easy to distinguish in cryo-EM (see Figure 4a). After INA treatment (flu + INA + UVA for 30 minutes), the general morphology of the influenza virus is maintained, with HA spikes still visible (Figure 4b). When the INA-treated sample is subjected to Triton X-100 (0.5%), however, the resulting suspension contains large viral aggregates that are not easily identifiable by cryo-EM as individual virions (Figure 4c). It appears that the viral membrane is disrupted by Triton treatment and therefore does not provide the sharp contrast needed in cryo-EM to see the HA spikes and general "round" nature of the virus. Interestingly, these viral aggregates are not seen in the detergent-treated control samples (flu + Triton alone, data not shown) where INA + UVA irradiation was not used, indicating here again that the INA treatment of the virus provides some detergent resistance in the viral suspension. To further probe the nature of these viral aggregates in cryo-EM, immunogold labeling studies were performed using an anti-HA antibody known to neutralize the virus [17,18]. These aggregates were shown to contain the antibody labeled proteins from the labeled virus (Figure 4d), which indicates that the aggregates contain influenza proteins, and potentially fragments of intact virus. An additional control showed that the binding of the immunogold was not due to non-specific interactions of the secondary antibody (Figure 4e). This image also shows that the aggregates are in focus, as noted by the sole immunogold particle located outside the boundary of the aggregate.

Both the IP data and cryo-EM data combined indicate that after Triton treatment, epitopes that are recognized by neutralizing anti-HA antibodies are still intact. Furthermore, the treatment results in a detergent resistant fraction, which when visualized by cryo-EM, appears as an aggregate of proteins. Since the membranes appear damaged and these clumps have a high electron density, it is uncertain whether these aggregates are clumps of virions missing portions of their membranes, such as lipids, (therefore the sharp contrast in electron density is no longer present) or if they are aggregates of pieces of the virus. With either scenario, the end result is that the viral proteins exhibit noticeable differences in their solubility after Triton treatment when INA + UVA irradiation is used versus control. This difference in solubility can be used towards the generation of a novel orthogonally inactivated vaccine strategy [10].

## Conclusions

In conclusion, we have shown that influenza virus can be successfully inactivated using both INA + UVA treatment and detergent treatment (Triton X-100). Additionally, with prolonged UVA irradiation (30 minutes) of influenza in the presence of INA, there is an increase in the detergent resistant (pelletable) fraction of the transmembrane protein HA2, with preservation of neutralization epitopes as characterize by immunoprecipitation and cryo-EM studies. All this, combined with previous results for HIV-1, indicate that our novel method for orthogonal inactivation with detergent resistance offers a unique and widely applicable route towards the generation of a new class of inactivated vaccines [10]. Further studies are needed to expand this method to other viruses and to in vivo studies to assess the effectiveness of these viral preparations versus the standard split virus vaccines.

## Materials and methods

### Reagents and cells

1,5-iodonaphthylazide (INA) was custom-synthesized by Biotium (Hayward, CA). Influenza stocks (for X31 and PR8 virus) were obtained from Charles River Laboratories (CRL) with the strains and lot numbers as specified in each method below. The following reagents were obtained through the NIH Biodefense and Emerging Infections Research Resources Repository ("BEI resources"), NIAID, NIH: Monoclonal Anti-Influenza A Virus HA2, Clone RA5-22 (produced in vitro), NR-4539 ("anti-HA2"); Polyclonal Anti-Influenza Virus H1 (H0) Hemagglutinin (HA), A/Puerto Rico/8/34 (H1N1), (antiserum, Goat), NR-3148 ("goat anti-HA", used in immunoprecipitation studies). The mouse anti-nucleoprotein ("anti-NP", A/New Caledonia/20/99, H1N1) antibody was purchased from eEnzyme (cat# MIA-NP-108). The mouse anti-HA tag antibody (cat# ab16918, recognizes a sequence in hemagglutinin from X31 virus and not PR8) was purchased from AbCam. Secondary antibody used for Western blot analysis was Alexafluor680 goat anti-mouse IgG (Invitrogen). Triton X-100 (Surfact-Amps, ampules of 10% solution in water, cat#PI-28314) was used for the detergent treatment of viruses. Sucrose (RNase-free, cat#S9378) was obtained from Sigma-Aldrich. Madin Darby Canine Kidney (MDCK) cells were obtained from the American Type Culture Collection (ATCC, cat # CCL-34), and were grown in MEM (ATCC, cat# 30-2003) containing 10% heat-inactivated fetal bovine serum (FBS, Invitrogen). TPCK-trypsin, used for the MDCK assay, was purchased from Pierce (cat#20233). Magnetic protein G Dynabeads (cat#100.03D) were purchased from Invitrogen, and protease inhibitor cocktail tablets (Complete Mini Protease Inhibitor, cat#11836170001) were purchased from Roche Applied Science. Avicel^® ^microcrystalline cellulose (RC-581) used for the MDCK plaque assay was kindly provided by FMC Biopolymer (Newark, DE).

### INA and detergent treatment of influenza

The Influenza used for the detergent treatment studies was purified Influenza A/PR/8/34 (PR8, H1N1) virus purchased from Charles River Laboratories (CRL), cat#10100374, 2 mg/mL total protein, batch#4XP100812 (lot tested by CRL and had an HA titer of 1:65,536 per 0.05 mL, and an egg infectious dose (EID50) of 10^9.5^/mL). The concentrated stock (2 mg/mL total protein) was thawed at room temperature and handled on ice immediately following thawing. The stock was diluted to 0.5 mg/mL total protein in PBS and treated with 100 micromolar INA according to similar published procedures [8,10]. In brief, INA was added from a 8 mM stock solution in DMSO for a final concentration of 100 micromolar in the flu suspension. The suspension was vortexed briefly and UVA irradiated for either 15 or 30 minutes, as specified in the results section.

For samples requiring subsequent detergent treatment, Triton X-100 was added (10 uL) from 10x Triton stock solutions added to the flu suspension (90 uL) for the final percent Triton as specified. Detergent treated samples were incubated at either room temperature (for MDCK infectivity studies only) or 37°C (MDCK and sucrose cushion pelleting studies) for one hour with vortex mixing every 15 minutes.

### Plaque assay for detergent-treated influenza

The influenza used for the MDCK assay was purified Influenza A/Aichi/68 (X31, H3N2) virus purchased from Charles River Laboratories (CRL), cat#490715, 2 mg/mL total protein, lots#4XAAH080627 and lot#4XAAH021203A. The lot number ending in 0627 was tested by CRL and had an HA titer of 1:4,194,304 per 0.05 mL, and an egg infectious dose (EID50) of 10^9.7^/mL. The lot number ending in 1203A had an HA titer of 1:16,384 per 0.05 mL, and an egg infectious dose (EID50) of 10^6.7^/mL.

The influenza was diluted to 0.5 mg/mL total protein in PBS just prior to detergent treatment. Any remaining stock was refrozen at -80°C at the original 2 mg/mL concentration.

Subsequent experiments with the remaining 2 mg/mL stock was considered to have gone through one "freeze/thaw" cycle and are labeled as such in the text. Stock solutions (10x) of triton X-100 were made in PBS; for example 2% stock was made and used for a final dilution of 0.2% triton in the viral suspension. To 180 microliters of the 0.5 mg/mL viral suspension was added 20 microliters of the appropriate 10x Triton stock. The samples either sat at room temperature or were placed into a 37°C incubator for 1 hour, with vortex mixing every 10 minutes to prevent settling of the virus suspension. After 1 hour, all samples were held at room temperature during dilution and addition to the plates containing the MDCK cells.

A plaque assay was used to determine the infectivity of influenza before and after detergent treatment. The assay was performed according to previously published procedures, using and Avicel overlay method [19,20]. In brief, MDCK cells were grown to confluency in 12-well plates. After washing the growth media (MEM with 10% FBS) from the cells using PBS, virus (either detergent-treated or control) was added to duplicate wells in MEM (without FBS) with TPCK-trypsin in a total volume of 300 microliters per well. Dilutions of 10^-1 ^and 10^-2 ^of the detergent-treated virus suspensions were added to the appropriate wells on the plate, and 10^-4 ^to 10^-7 ^dilutions were added for the controls. In the case where 0.3% - 0.5% Triton X-100 was used to treat the virus, either 10^-2 ^to 10^-3 ^were plated instead, or the initial detergent-treated stock was diluted by a factor of two in MEM without FBS, to overcome the toxicity of the residual Triton to the cells. These modifications to the dilutions were accounted for in the calculations of plaque-forming units (PFU/mL) used to determine the log reduction in infectivity after detergent treatment. After adding the samples to the wells, the plates were incubated at 37°C for 1 hour. Subsequently, the media was removed and was replaced with fresh media containing MEM, HEPES, TPCK-trypsin, and 1.2% Avicel, and plates were incubated at 37°C for 48 hours. After incubation, the media and Avicel overlay were removed and each well was gently washed with PBS to remove residual Avicel. Cells were fixed using 70% Ethanol and stained using crystal violet to visualize the plaques as regions of missing cells on a lawn of purple-stained cells.

### Hemagglutination assay

Hemagglutination assay was performed according to previously published procedures [19]. Two separate lots of X31 virus were tested; CRL lot numbers ending in -0627 and -1203A as were used for the MDCK plaque assay described above. In brief, red blood cells were purified via centrifugation from human blood that was collected from donors in sodium citrate tubes. Isolated red blood cells (RBC's) were suspended for a final concentration of ~1% in phosphate buffered saline (PBS). To each well in a v-bottom 96-well plate was added 50 microliters of virus (control or INA (100 micromolar) + UVA treated) in PBS, diluted two-fold across the plate. To each well was then added 50 uL of 1% human RBC's. Plate was gently agitated to mix contents and allowed to sit for one hour at room temperature before reading. The hemagglutination unit (HAU) titer was determined from the highest dilution that showed hemagglutination (where the RBC's were held in solution by the virus).

### Sucrose cushion and pelleting of detergent-treated virus with western blot analysis

Both detergent-treated and control samples were ultracentrifuged through a sucrose cushion to separate out the soluble and insoluble fractions, and analyzed similar to previously published procedures [10]. 200 uL of 30% sucrose (in PBS, verified using a Bausch and Lomb refractometer) was placed into a polycarbonate ultracentrifuge tube (Beckman #343776, 8 mm × 34 mm). To this, 90 uL of detergent-treated flu sample was carefully added (undiluted) on top of the sucrose cushion with caution to maintain the phase boundary. The samples were then ultracentrifuged (Optima TLX tabletop ultracentrifuge) in a fixed-angle rotor (TLA 120.1) at 58,000 rpm for one hour at 4°C. Immediately after centrifugation, the supernatant (290 uL total) was carefully removed and added to Laemmli reducing sample buffer in a microfuge tube. To the remaining pellet, still in the centrifuge tube, was added 290 uL of PBS and sample buffer. Pellets were very sticky after centrifugation so care was needed to resuspend them in the sample buffer by aspirating gently with a pipette tip and pipettor. The entire centrifuge tube, with pellet solution, was placed into a larger 2 mL microfuge tube with a lid. The samples were heated at 70°C for 15-20 minutes and SDS-PAGE under denaturing and reducing conditions was run (Invitrogen, Tris-glycine gels, 10-well, 4-20%). The gels were blotted onto nitrocellulose and probed via Western blot using MAbs for the proteins of interest, and Alexafluor 680 conjugated Mouse IgG secondary antibody for IR readout and integration using an Odyssey imaging system and software. Separate lanes for the pellet (P) solution and supernatant (S) solution were run for each sample. Integration of each of these lanes was done using the Odyssey software and each lane was selected to determine the raw integrated intensity of the entire lane. The entire lane was selected to include all the protein aggregates that contained the probed protein of interest, and the entire lanes were integrated in the controls as well for direct comparison. The "percent in pellet" (%P) was then determined using these raw integrated intensities as follows: %P = P/(P + S), where P and S are the respective integrations of the pellet and supernatant lanes for one sample. Separate gels were run for each protein; gels were not reprobed. Each treatment was triplicate via three separate experiments starting with fresh viral stock each time. P-values were calculated using these triplicate in a two-tailed student's T-test with unequal variances, with significant values (*P *< 0.05).

### Immunoprecipitation of pelleted influenza proteins

For immunoprecipitation studies, the purified influenza virus stock from CRL was influenza A/PR/8/34 (PR8, H1N1), cat#10100374, 2 mg/mL total protein, batch#4XP110510 (lot tested by CRL and had an HA titer of 1:1,048,576 per 0.05 mL, and an egg infectious dose (EID50) of 10^10.0^/mL). Influenza samples were diluted to 0.5 mg/mL in PBS, treated with INA (100 micromolar) plus UVA for 30 minutes, treated with detergent (37°C for 1 hour with vortex mixing every 15 minutes) and pelleted thru sucrose as specified above. Mock-treated (detergent only) samples were also prepared in similar fashion. For immunoprecipitation studies, the supernatant was discarded and 90 uL of 1% Triton X-100 in PBS with protease inhibitors was added to the pellet. The pellet was gently resuspended, transferred to a microfuge tube, and 10 microliters of Goat anti-HA antibody (BEI resources) was added to each sample. Samples were incubated overnight with rotation at room temperature, covered from light. The following morning, Dynabeads (prewashed in two volumes of 1% Triton X-100 in PBS) were added (33.3 uL) to each sample. Samples were rotated again at room temperature for 45 minutes. A magnet was applied, supernatant was removed and beads were washed with 2 volumes of 1% Triton X-100 in PBS (with protease inhibitor) and two volumes of PBS, with resuspension of the beads and magnet application between washing steps. After the final wash, the beds were transferred to a new microfuge tube, a magnet was applied, PBS was removed, and 60 uL of 1x Laemmli sample buffer (reducing) was added to each sample. The samples were heated at 70°C for 15-20 minutes and SDS-PAGE under denaturing and reducing conditions was run (Invitrogen, Tris-glycine gels, 15-well, 4-20%). The gels were blotted onto nitrocellulose and probed via Western blot using mouse anti-HA2 antibody (BEI resources), and Alexafluor 680 conjugated Mouse IgG secondary antibody for IR readout and integration using an Odyssey imaging system and software.

### Cryo-transmission electron microscopy analysis

For non-immunogold EM studies, the influenza viral lot ending in #812 from CRL was used. For the immunogold EM studies, the purified PR8 influenza virus stock from CRL batch#4XP110510 was used. Influenza stocks were diluted to 0.5 mg/ml total protein in PBS. Half was treated with INA (100 micromolar, as previously described) then UVA irradiated for 30 minutes. The remainder was left untreated as a control. For the immunogold labeled samples: to 100 uL of each was added a 1:25 dilution of primary antibody (BEI resources goat antiserum, anti-H0) and samples were incubated for 1 hour at room temperature. Control samples were also prepared that omitted the primary antibody and used PBS in its place to assess for non-specific adsorption of the secondary immunogold antibody. Following this incubation, samples (90 uL) were pelleted through a 200 uL 30% sucrose pad (as described above). Pellets were gently resuspended in 90 uL of 1 molar HEPES buffer, transferred to a fresh microfuge tube to which 10 uL of either Triton X-100 (5%) or PBS was added, for a final detergent concentration of 0.5% and 0% respectively. Microfuge tubes were incubated at 37°C for 1 hour with vortexing at t = 0, 30 min and 1 hour. After 1 hour, 10 microliters of secondary immunogold antibody (15 nm rabbit anti-goat IgG, Aurion) was added to all samples, and incubated at room temperature for an additional 30 minutes. Samples were then stored overnight at 4°C. The following day, fixative (8% formaldehyde in 1 M HEPES buffer) was added 1:1 to each sample 30 minutes prior to freezing the samples on grids for cryo-EM analysis. For the non-immunogold samples: influenza was prepared as described with the omission of the primary, secondary antibody and ultracentrifugation steps. For these samples, triton (10 microliters of 10x) was added to the diluted +/- INA-treated samples (90 microliters) and incubated at 37°C for 1 hour with periodic vortex mixing as described above. Fixative was added immediately following this incubation and cryo-EM was performed. For cryo-EM, 4 μL of sample were blotted onto glow-discharged holey carbon grids R2/2 (Quantifoil) and vitrified in a Vitrobotcryostation (FEI). Images were recorded with a T20 microscope (FEI) at 200 kV on an Eagle CCD camera (FEI).

## Competing interests

The authors declare that they have no competing interests.

## Authors' contributions

JMB performed and helped to design the detergent resistance studies and associated assays. UB performed the cryo-TEM analysis. YR, MV, RB conceived of the study, and participated in its design and coordination. All authors read, helped with revisions, and approved the final manuscript.

## Supplementary Material

Additional file 1 **S1 Amount of Triton required to remove infectious material from Influenza X31 as measured using an MDCK plaque assay**. (expansion of data shown in Table 2 in text). Click here for file
