# Supplementary material for: Orthogonal inactivation of influenza and the creation of detergent resistant viral aggregates: towards a novel vaccine strategy
Source: Virol J. 2012 Mar 26;9:72. doi: 10.1186/1743-422X-9-72 (PMC3353219; doi:10.1186/1743-422X-9-72)
Supplement: Additional file 1 — S1 Amount of Triton required to remove infectious material from Influenza X31 as measured using an MDCK plaque assay. (expansion of data shown in Table 2 in text). [file 1743-422X-9-72-S1.PPTX]

## Slide 1
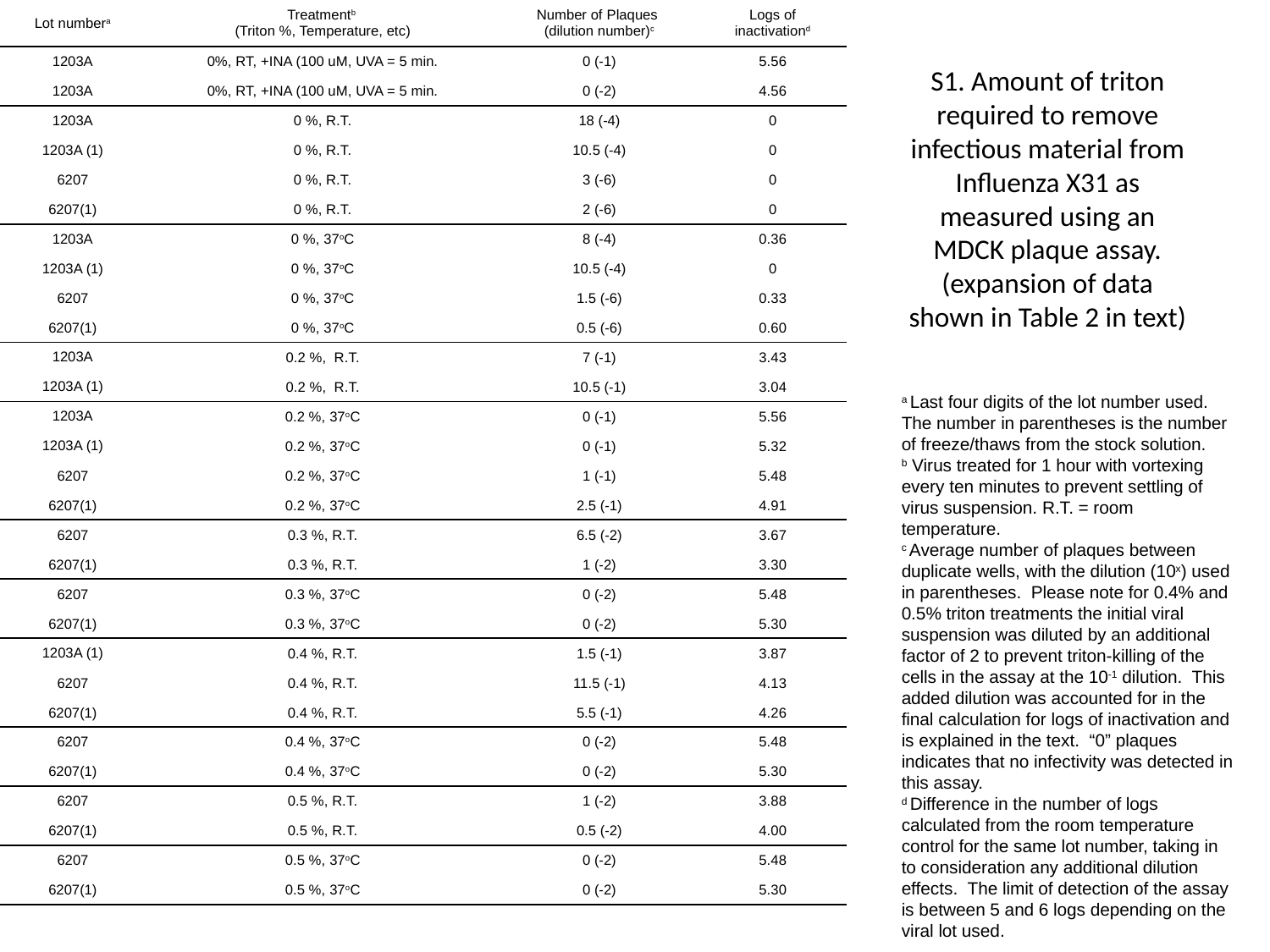

| Lot numbera | Treatmentb (Triton %, Temperature, etc) | Number of Plaques (dilution number)c | Logs of inactivationd |
| --- | --- | --- | --- |
| 1203A | 0%, RT, +INA (100 uM, UVA = 5 min. | 0 (-1) | 5.56 |
| 1203A | 0%, RT, +INA (100 uM, UVA = 5 min. | 0 (-2) | 4.56 |
| 1203A | 0 %, R.T. | 18 (-4) | 0 |
| 1203A (1) | 0 %, R.T. | 10.5 (-4) | 0 |
| 6207 | 0 %, R.T. | 3 (-6) | 0 |
| 6207(1) | 0 %, R.T. | 2 (-6) | 0 |
| 1203A | 0 %, 37oC | 8 (-4) | 0.36 |
| 1203A (1) | 0 %, 37oC | 10.5 (-4) | 0 |
| 6207 | 0 %, 37oC | 1.5 (-6) | 0.33 |
| 6207(1) | 0 %, 37oC | 0.5 (-6) | 0.60 |
| 1203A | 0.2 %, R.T. | 7 (-1) | 3.43 |
| 1203A (1) | 0.2 %, R.T. | 10.5 (-1) | 3.04 |
| 1203A | 0.2 %, 37oC | 0 (-1) | 5.56 |
| 1203A (1) | 0.2 %, 37oC | 0 (-1) | 5.32 |
| 6207 | 0.2 %, 37oC | 1 (-1) | 5.48 |
| 6207(1) | 0.2 %, 37oC | 2.5 (-1) | 4.91 |
| 6207 | 0.3 %, R.T. | 6.5 (-2) | 3.67 |
| 6207(1) | 0.3 %, R.T. | 1 (-2) | 3.30 |
| 6207 | 0.3 %, 37oC | 0 (-2) | 5.48 |
| 6207(1) | 0.3 %, 37oC | 0 (-2) | 5.30 |
| 1203A (1) | 0.4 %, R.T. | 1.5 (-1) | 3.87 |
| 6207 | 0.4 %, R.T. | 11.5 (-1) | 4.13 |
| 6207(1) | 0.4 %, R.T. | 5.5 (-1) | 4.26 |
| 6207 | 0.4 %, 37oC | 0 (-2) | 5.48 |
| 6207(1) | 0.4 %, 37oC | 0 (-2) | 5.30 |
| 6207 | 0.5 %, R.T. | 1 (-2) | 3.88 |
| 6207(1) | 0.5 %, R.T. | 0.5 (-2) | 4.00 |
| 6207 | 0.5 %, 37oC | 0 (-2) | 5.48 |
| 6207(1) | 0.5 %, 37oC | 0 (-2) | 5.30 |
S1. Amount of triton required to remove infectious material from Influenza X31 as measured using an MDCK plaque assay. (expansion of data shown in Table 2 in text)
a Last four digits of the lot number used. The number in parentheses is the number of freeze/thaws from the stock solution.
b Virus treated for 1 hour with vortexing every ten minutes to prevent settling of virus suspension. R.T. = room temperature.
c Average number of plaques between duplicate wells, with the dilution (10x) used in parentheses. Please note for 0.4% and 0.5% triton treatments the initial viral suspension was diluted by an additional factor of 2 to prevent triton-killing of the cells in the assay at the 10-1 dilution. This added dilution was accounted for in the final calculation for logs of inactivation and is explained in the text. “0” plaques indicates that no infectivity was detected in this assay.
d Difference in the number of logs calculated from the room temperature control for the same lot number, taking in to consideration any additional dilution effects. The limit of detection of the assay is between 5 and 6 logs depending on the viral lot used.
